# Electrospinning Pullulan Fibers from Salt Solutions †

**DOI:** 10.3390/polym9010032

**Published:** 2017-01-22

**Authors:** Ran Li, Peggy Tomasula, Ana Margarida Moreira de Sousa, Shih-Chuan Liu, Michael Tunick, Kevin Liu, Linshu Liu

**Affiliations:** 1 Dairy and Functional Foods Research Unit, Eastern Regional Research Center, Agricultural Research Service, US Department of Agriculture, 600 E. Mermaid Lane, Wyndmoor, PA 19038, USA; ran.li2@wsu.edu (R.L.); peggy.tomasula@ars.usda.gov (P.T.); Ana.M.Sousa@saint-gobain.com (A.M.M.d.S.); michael.tunick@ars.usda.gov (M.T.); 2 State Key Laboratory of Separation Membranes and Membrane Processes, Tianjin Polytechnic University, #399 Binshuixi Road, Xiqing District, Tianjin 300387, China; 3 School of Health Diet and Industry Management, Chung-Shan Medical University and Department of Nutrition, Chung Shan Medical University Hospital, No. 110, Sec. 1, Jianguo N. Rd., South District, Taichung 402, Taiwan; 4 National Center for Agricultural Utilization Research, Agricultural Research Service, U.S. Department of Agriculture, 1815 N. University Street, Peoria, IL 61604, USA; kevin.liu@ars.usda.gov

**Keywords:** ultrafine fibers, rheology, morphology, pullulan

## Abstract

There is an increasing interest in applying the technology of electrospinning for making ultrafine fibers from biopolymers for food-grade applications, and using pullulan (PUL) as a carrier to improve the electrospinnability of proteins and other naturally occurring polyelectrolytes. In this study, PUL solutions containing NaCl or Na_3_C_6_H_5_O_7_ at different concentrations were electrospun. The inclusion of salts interrupted the hydrogen bonding and altered solution properties, such as viscosity, electric conductivity, and surface tension, as well as physical properties of fibers thus obtained, such as appearance, size, and melting point. The exogenous Na^+^ associated to the oxygen in the C6 position of PUL as suggested by FTIR measurement and was maintained during electrospinning. Bead-free PUL fibers could be electrospun from PUL solution (8%, *w/v*) in the presence of a 0.20 M NaCl (124 ± 34 nm) or 0.05 M Na_3_C_6_H_5_O_7_ (154 ± 36 nm). The further increase of NaCl or Na_3_C_6_H_5_O_7_ resulted in fibers that were flat with larger diameter sizes and defects. SEM also showed excess salt adhering on the surfaces of PUL fibers. Since most food processing is not carried out in pure water, information obtained through the present research is useful for the development of electrospinning biopolymers for food-grade applications.

## 1. Introduction

Pullulan (PUL) is an extracellular polysaccharide produced by yeasts. It is a linear glucan consisting of three glucose units connected by α-1,4 glycosidic bonded maltotriose that are linked via α-1,6 glycosidic linkage. In some cases, depending on the biosynthetic origins, maltotetraose subunits may substitute predominant maltotriose subunits [1]. Commercially available PUL is an odorless, tasteless, white colored powder that is highly water soluble, non-toxic, stable to most metals, and resistant to changes in temperature and solution pH [2,3,4,5,6,7,8,9,10]; PUL is also inert to mammalian amylases and possesses prebiotic properties, thus it is used as low calorie, dietary fibers in health foods and functional food [2,10,11,12,13,14,15]. Recently, in the attempt to expand food resources and develop new food formulations, PUL fibers and fibrous mats were electrospun in combination with caseinates and pectin in our laboratory [16,17], along with several other food-grade biopolymers from the groups of protein, polysaccharides, and lipids [18].

Fibers and fibrous mats on the submicron or nano scale possess several advantages: huge surface area to volume ratios, accessibility and flexibility in surface modifications, as well as excellent mechanical properties such as tensile strength and modulus. Thus they have a huge potential for food, biomedical, and engineering applications [16,18,19]. In comparison to synthetic polymers, most biopolymers are relatively difficult to electrospin. One of the technical challenges is that many biopolymers tend to form strong hydrogen bonds leading to gel formation that impacts negatively on electrospinning [20]. The approach that has been popularly adapted to tackle this issue is to use a synthetic polymer, such as poly(ethylene oxide) or poly(vinyl alcohol) to facilitate molecular entanglement for inducing fiber formation. To exclude the use of synthetic, non-food polymers and non-aqueous solvents critical for food-grade applications, PUL has been proposed as an alternative and tested in our laboratory for electrospinning food grade biopolymers [16,17,21], and evaluated for the correlation between the polymer’s electrospinnability and solvent properties [16,17,22,23]. Process parameters, such as voltage, temperature, distance between capillary and screen, etc., were also evaluated case by case, since these elements were instrument-specific, although a set of essential principles were established [19,24].

In the present study, PUL fibers were electrospun from aqueous solutions containing different concentrations of sodium chloride and sodium citrate with fixed operational conditions, by which bead-free PUL fibers could be obtained from salt-free aqueous solutions. We investigated the effect of salts on fiber formation and fiber characteristics, in terms of morphology, size, salt uptake, and crystallinity. The research is essential for electrospinning biopolymer fibers for potential food grade applications, since most food processing is not carried out in pure water; in addition, most food components form charged ions upon dissolving.

## 2. Materials and Methods

### 2.1. Materials

PUL was purchased from TCI America (Portland, OR, USA). Sodium chloride (NaCl) and sodium citrate (Na_3_C_6_H_5_O_7_) and other chemicals were from Sigma-Aldrich (St. Louis, MO, USA). Deionized water (D.I. water) was prepared using a Barnstead E-pure water system (Dubuque, IA, USA) and used to prepare all aqueous solutions.

### 2.2. Methods

#### 2.2.1. Electrospinning

PUL aqueous solutions were prepared by dissolving the polysaccharide with D.I. water or solutions of NaCl or Na_3_C_6_H_5_O_7_ at predetermined concentrations on weight/volume ratios. All PUL solutions were degassed by storing at 4 °C overnight prior to use. The electrospinning was performed on a NaBond nanofiber electrospinning unit (NaBond Technologies, Hong Kong) equipped with a syringe pump, a high voltage generator, and a grounded rotating cylinder receptor. A schematic presentation of the electrospinning set-up could be found in our previous publication [25]. After several preliminary trials the electrospinning conditions were fixed at: distance from the needle tip to the receptor, 12 cm; flow rate, 1.5–2.0 mL/h; voltage, 18–20 kV; temperature, 22 ± 2 °C; and drum rotating rate, 40 rpm. These conditions ensured perfect polymer jets (continuous and free of droplets) for 15% PUL aqueous solution (*w/v*). To focus on the effect of salts on PUL electrospinnability, all PUL formulations were electrospun thereafter under these fixed conditions.

#### 2.2.2. Solution Analysis

Prior to electrospinning, PUL solutions were characterized for rheology, electric conductivity, and surface tension.

The viscosities of PUL solutions were investigated using a rheometer equipped with a 30 mm inside diameter cup and a 28 mm outside diameter bob (AR 2000; TA Instruments, New Castle, DE, USA). Flow curves were built from apparent viscosity vs. shear rate data collected at room temperature (22 ± 2 °C) with the shear rate changing from 1 to 150 s^−1^.

The electrical conductivities of the PUL solutions, sodium salt solutions and their mixtures, were measured using a conductivity meter (IQ270G; Scientific Instruments, Loveland, CO, USA). The surface tensions of these solutions were determined using a Fisher Scientific^TM^ Surface Tension Apparatus (ring diameter, 1.905 cm; Fisher Scientific, Suwanee, GA, USA).

All measurements were conducted at room temperature, each sample was examined for 5–10 times, and an average was taken.

#### 2.2.3. Fiber Characterization

Resultant fibers and mats were characterized by high-performance size-exclusion chromatographic (HPSEC) for molecular properties, scanning electron microscopy (SEM) for morphology, Fourier transform infrared spectroscopy (FTIR) for chemical structure, and differential scanning calorimetry (DSC) for thermal properties.

Aqueous solutions of the original PUL and PUL fibers were made by dissolution of the polymers in 0.05 M NaNO3 (1 mg/mL). HPSEC was performed on a Waters HPSEC (Waters Inc., Milford, MA, USA) system equipped with a differential pressure viscometer (ViscoStar model; Wyatt Technology, Santa Barbara, CA, USA), a Waters 2410 differential refractometer (RI), and two PL-Aquagel size exclusion columns (OH-60 and OH-40) in series and an auto sampler (717 Plus Auto Injector, Waters). Molecular properties of electrospun PUL in terms of weight average molecular weight (*M*_w_), root mean square radius of gyration (*R*_gz_), and second Virial coefficient (*a*) were collected by the method described previously [26].

SEM was conducted on a scanning electron microscope (FEI, Hillsboro, OR, USA) after coating the fibers with a thin film of gold. Micrographs were taken in the high-vacuum/secondary electron imaging modes at an accelerating voltage of 10 kV. Fiber diameter sizes were measured randomly on 100 fibers per sample using the image analysis software XT Document (FEI Corp, Hillsboro, OR, USA), which was pre-installed in the computer, to construct a diameter histogram.

FTIR spectra were recorded on a Thermo Nicolet Nexus 470 FTIR system (Madison, WI, USA) coupled with Smart ARK accessory for liquid samples in a scanning range of 650–4000 cm^−1^ for 32 scans at a spectra resolution of 4 cm^−1^. Solid samples were recorded on the same FTIR system coupled with Smart Orbit accessary.

DSC experiments were carried out using a DSC 2910 (TA Instruments). Approximately 5–10 mg of each sample was weighed and sealed in a 40 µL aluminum crucible. All measurements were operated under nitrogen atmosphere from 25 to 250 °C at the heating rate of 10 °C/min.

PUL powder without submission for electrospinning was used as the control in all measurements.

#### 2.2.4. Statistical Analysis

The Shapiro-Wilk W test adapted to large sample sizes by Royston was used for testing normality (*p* < 0.05) of the fiber diameter distributions. Means of fiber sizes following the normality and homogeneity of variance requirements were compared using the Tukey test (*p* < 0.05) while the Mann-Whitney test (*p* < 0.05) was used for comparing means of fiber diameters with non-normal distributions. The statistical analysis was performed using the software Statistica 8.0 (StatSoft Inc., Tulsa, OK, USA).

## 3. Results and Discussion

### 3.1. Effect of Sodium Salts on the Electrospinnability and Morphologies of PUL Fibers

**Figure 1:**
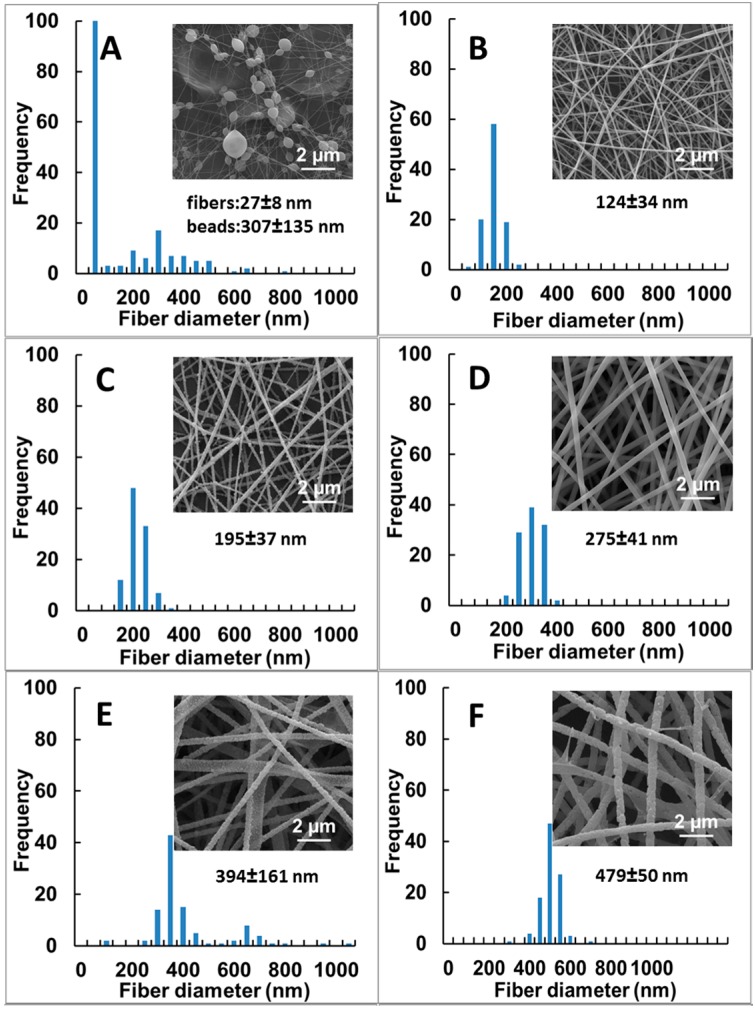

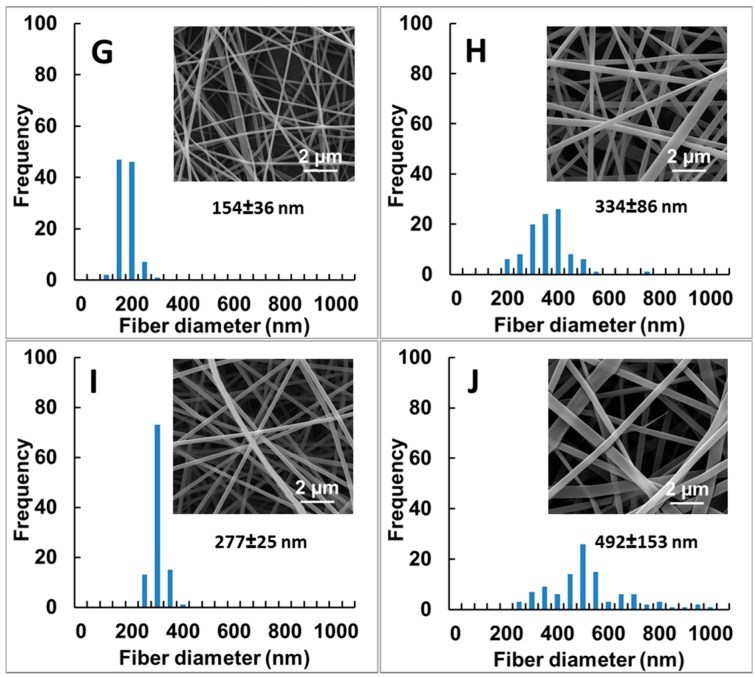
Size and size distribution of pullulan (PUL) fibers electrospun from PUL solutions with different types and concentrations and salts shown in the respective SEM image: 8% PUL without salt (**A**); and with NaCl at 0.20 M (**B**) and 1.0 M (**C**); 15% PUL without salt (**D**); and with NaCl at 2.0 M (**E**) and 5.0 M (**F**); 8% PUL with Na_3_C_6_H_5_O_7_ at 0.05 M (**G**) and 0.50 M (**H**); 15% PUL with Na_3_C_6_H_5_O_7_ at 0.05 M (**I**) and 0.50 M (**J**). Magnification is 25,000×.

PUL fibers and fibrous mats were electrospun from 8% and 15% PUL solutions with various amounts of NaCl or Na_3_C_6_H_5_O_7_, respectively, added. The resultant fibers were examined by SEM. From the respective micrographic images, quantitative analysis of fiber diameter and diameter size distribution was conducted. The results are summarized in Figure 1, revealing the impact of salt concentration on fiber formation and fiber morphology. Beaded fibers were obtained from neat PUL at 8% (Figure 1A); the inclusion of NaCl at the concentrations of 0.01 and 0.10 M did not alter fiber appearances (data not shown) but bead-free fibers with an average diameter of 124 ± 34 nm were electrospun by the inclusion of NaCl at 0.20 M to the same PUL solution (Figure 1B). The further increase in NaCl concentration increased the fiber size (Figure 1C; 195 ± 37 nm), which became normally distributed (*p* > 0.05). The increase in PUL concentration from 8% to 15% resulted in the transition of beaded-fiber to bead-free fiber (Figure 1D) that is in consistent with our previous finding [16]. In addition, as shown in Figure 1D–F, the fiber diameter increased (*p* < 0.01) from 275 ± 41 to 394 ± 161 and 479 ± 50 nm, as the 15% PUL migrated from salt free solution to 2.0 and 5.0 M NaCl solutions, respectively. The inclusion of Na_3_C_6_H_5_O_7_ to the PUL solutions also resulted in thicker fibers produced (Figure 1G–J), but much less sodium citrate than sodium chloride was required in solution to attain a similar effect on fiber size and morphology. At a concentration as low as 0.05 M, the inclusion of Na_3_C_6_H_5_O_7_ to 8 wt % PUL eliminated bead formation. This could be attributed to the higher ionic strength of the Na_3_C_6_H_5_O_7_ solution.

**Figure 2:**
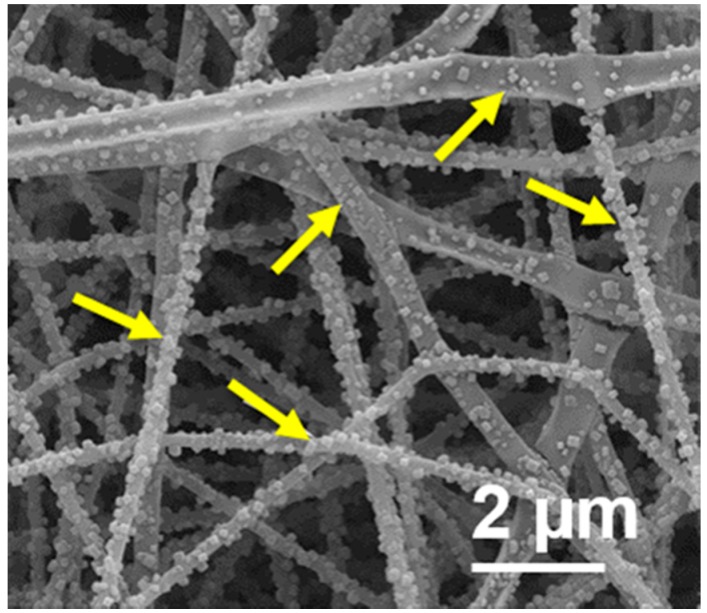
SEM image of PUL fiber (15% PUL, 1.0 M NaCl) at high magnification. Crystals of sodium chloride are observed on the fiber surfaces. Arrows indicate salt crystals.

Furthermore, it was observed that the increase in fiber diameter (*p* < 0.01) from lower (Figure 1E,I) to higher salt concentrations (Figure 1F,J) was accompanied by the formation of defects on the fiber surfaces, emergence of a flat appearance, and observation of salt crystals (Figure 2). Without salt inclusion, the electrospun fibers showed a smooth and continuous appearance and were even and thin (Figure 1D). The morphology of the electrospun PUL fibers appeared to be altered by salt concentration. The salt-dependent morphology was also seen over all samples electrospun from 8% of PUL solutions containing more than 2.0 M NaCl (data not shown).

To further explore the role of NaCl and Na_3_C_6_H_5_O_7_ in PUL fiber electrospinning, we investigated the PUL-salt solution properties in terms of rheology, electrical conductivity, and surface tension. FTIR was used to investigate of the interactions between the sodium ions and the polysaccharide.

### 3.2. Effect of Sodium Salts on Solution Properties

**Figure 3:**
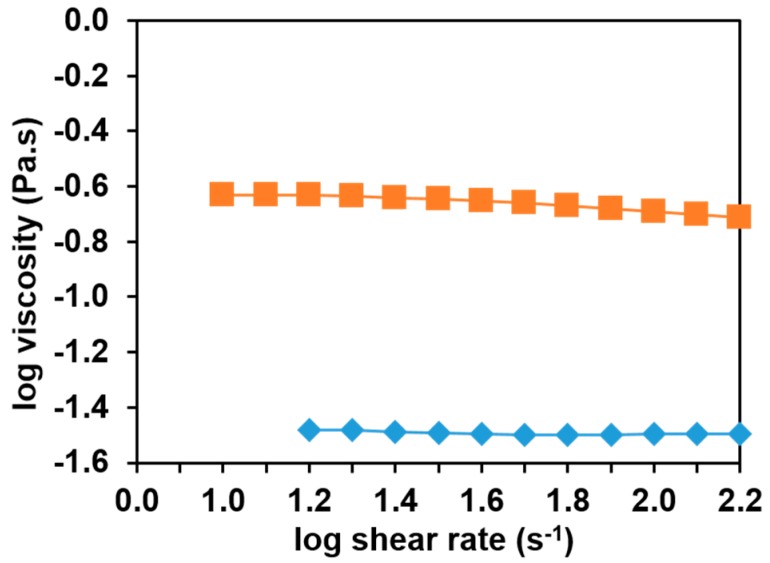
Logarithm of apparent viscosity of 15% (■) and 8% (♦) pullulan solutions as function of the logarithm of shear rate.

Figure 3 shows the logarithm of apparent viscosities of the two PUL solutions as a function of the logarithm of shear rate.

At the lower PUL concentration of 8%, the solution was a Newtonian fluid with apparent viscosity of around 0.03 Pa·s independent of shear rate. At the higher concentration of 15%, the PUL solutions showed shear thinning behavior, although the influence of shear rate on apparent viscosity was still very light. In the range of low shear rates, the apparent viscosity of the 15% PUL solution was about eight times higher than that of the 8% PUL (e.g., ~0.25 Pa·s at 15 s^−1^). This concentration-dependent shear rate vs. viscosity relations are in consistent with that reported in literature [23]. We then calculated the specific viscosity (η_sp_) of all PUL solutions by η_sp_ = (η_0_ − η_s_)/η_s_, where η_0_ is the solution zero shear rate viscosity and η_s_ is the solvent viscosity (water in the present experiments). The zero shear rate viscosities of all solutions were collected by extrapolating the shear rate curve to the lower end. Table 1 shows the specific viscosities of the two PUL solutions and their mixtures with various amounts and types of sodium salts.

The inclusion of NaCl or Na_3_C_6_H_5_O_7_ initially resulted in an increase of PUL solution viscosity. This was observed for both 8% and 15% PUL with NaCl from 0.20 to 2.0 M, and Na_3_C_6_H_5_O_7_ from 0.05 to 5.0 M. However, as salt concentration further increased, specific viscosity decreased (e.g., at 5.0 M NaCl for 15% PUL as shown in Table 1). It was reported that the inclusion of metal salts into locust bean gum solutions altered the solution viscosity [27], as we observed with PUL solutions in the present research. Presumably, the included metal ions disrupt the hydrated structures of the macromolecules associated by hydrogen bonding. The types of hydrogen bonding in PUL solutions include those between the polymer chains and water molecules, and those formed intra- and inter-polysaccharide chains. The interruption of hydrogen bonding between PUL and water reduces the viscosity of the solution. The interruption of hydrogen bonding within the PUL chains is relatively complicated. It may result in a decrease of viscosity of PUL, as chain–chain interactions are reduced. On the other hand, the interruption of inter- and intra-chain interactions result in an increase of the solubility of the macromolecules that is associated with the increase of hydroxyl bonding between the polysaccharide chains and water molecules, and an increase in solution viscosity can be anticipated. From Table 1, it seems that the addition of a small portion of metal salts into PUL solutions interrupted the hydrogen bonding mainly within the PUL macromolecular chains, releasing more flexible segments of the polysaccharide. Not only did solution viscosity increase, but the expansion of chain conformation also facilitated molecular entanglement. However, as more salts were added (i.e., at 5.0 M), a “salting out” effect occurred by which the water molecules were peeled from PUL chains, and the solution viscosity decreased (e.g., 15% PUL with 5.0 M NaCl in Table 1).

**Figure 4:**
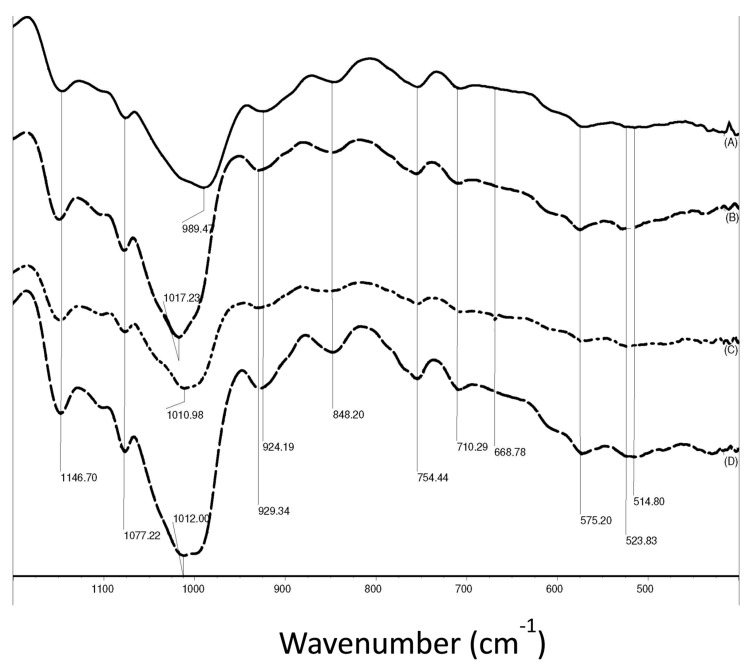
FTIR spectra of pullulan samples. From top to bottom: powders prior to electrospinning (**A**); pullulan fibers electrospun from solutions containing 2.0 M NaCl (**B**); 0.10 M NaCl (**C**); and 0.80 M NaCl (**D**), respectively.

We recorded the FTIR spectra of PUL samples electrospun from 8% PUL solutions with different NaCl contents. Spectra from representative pullulan samples are shown in Figure 4. The band seen at 848 cm^−1^ is the characteristic of the α–configuration of α–d-glucopyranose units. The absorption at 755 and 924 cm^−1^ demonstrated that the predominant linkages between glucose units were α(1,4) and α(1,6), which are two main linkages within PUL. Other features of the polysaccharide were also identified from FTIR examination, but not shown in the spectra: O–H stretch (3431–3435 cm^−1^), C–H stretch (2928–2929 cm^−1^), O–C–O stretch (1645 cm^−1^), C–O–H bend (1366–1368 cm^−1^), and C–O–C stretch (1154–1155 cm^−1^). It is worth noting that the band at 989 cm^−1^ associated with C–O–H bending vibration at the C6 position, indicating the stretch of the interchain interaction via hydrogen bonding for PUL, was found in the PUL powder samples. The absorption at 989 cm^−1^ was shifted to 1009, 1012, and 1016 cm^−1^ for PUL fibers electrospun from PUL solutions with NaCl concentrations at 0.10, 0.80, and 2.0 M, respectively. These shifts of C–O–H bending modes may originate from the association of Na^+^ ions and the oxygen at C6. The association of sodium ions with the –O– at the C6 position were further demonstrated by the band shift from 924 cm^−1^ that was found in the PUL powders to 929 cm^−1^ for the PUL fibers electrospun from its NaCl solutions. The association of oxygen with sodium ion found for electrospun PUL fibers is similar to that reported for the interactions in aqueous solutions for Na^+^-gellan gum [28], PUL with Cu^++^ complex [29], and Na^+^ with PEO [30]. From the FTIR measurements, it is suggested that the inclusion of Na^+^ ions in PUL solution interrupted the existing hydrogen bonding, and the resultant Na^+^ and oxygen associations were eventually maintained during electrospinning as H_2_O molecules were evaporated. This agrees well with the changes in solution properties that could lead to the changes in fiber morphology and fiber size discussed in previous sections (Figure 1 and Figure 2).

**Figure 5:**
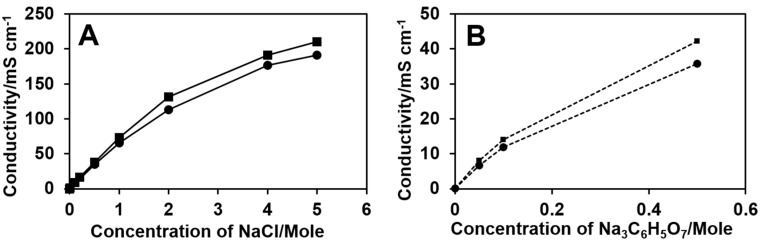
Electrical conductivity of PUL solutions 8% PUL (■) and 15% PUL (●) with different NaCl (**A**) and Na_3_C6H_5_O_7_ (**B**) content.

**Figure 6:**
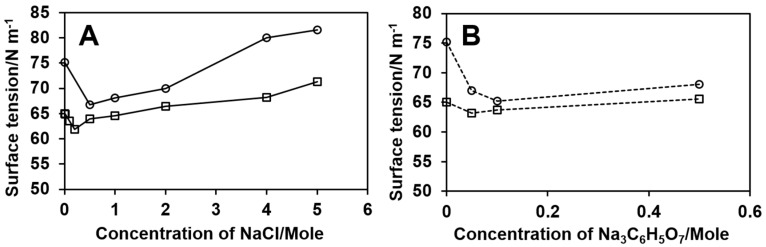
Surface tension of PUL solutions 8% PUL (□) and 15% PUL (○) with different NaCl (**A**) and Na_3_C6H_5_O_7_ (**B**) content.

The effect of salts on the electrical conductivity and surface tension of both 8% and 15% PUL solutions with various salts contents was also investigated. The 15% PUL solutions seemed slightly less conductive than 8% PUL solutions above 0.20 M salt content (Figure 5), and showed higher values of surface tension over the entire range of salt concentrations considered in this study (Figure 6). The electrical conductivity of PUL solutions increased with the increase in salt concentration (Figure 5). The values of the surface tensions of PUL solutions were low with the addition of a small portion of NaCl; then increased as more salts dissolved; however, over the ranges of 0.1–3.0 M NaCl, and 0.1–0.5 M Na_3_C_6_H_5_O_7_ the salt-PUL solutions possessed a lower surface tension than the neat PUL solution (Figure 6).

Thus, we hypothesized that the initial salt inclusion to 8% PUL solution appeared to favor the formation of fibers, because of the increase in solution viscosity (Table 1) that promoted the entanglement of PUL chains needed for fiber formation, and the decrease in surface tension that is recommended for the electrostatic force built up to pull the PUL drop as a continuous jet towards the drum. As shown in Figure 1A,B, 0.20 M NaCl in 8% PUL resulted in the conversion of beaded fibers to bead-free fibers. As the concentration of NaCl in 15% PUL solution further increased to 5.0 M, solution viscosity was lowered, and a remarkable increase in both electric conductivity and surface tesion of the solution was noted, which weakened the electrostatic forces that draw PUL pendant drops, resulting in large and flat-looking fibers, and defects in the fibers.

### 3.3. Effect of Sodium Salts on the Thermodynamic Property of Resultant Fiber

FTIR spectroscopy provided qualitative information on the binding sites of salt ions on the PUL macromolecules that can be complemented by thermodynamic characterization either by heat flow thermometry [31,32,33] or by DSC as conducted in the present research.

**Figure 7:**
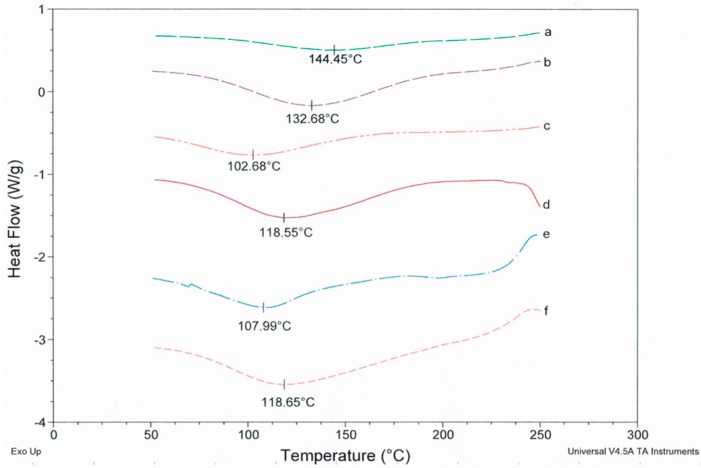
DSC scanning results of (**a**) pullulan powders; (**b**) electrospun pullulan fibers without salts; (**c**) with 2.0 M NaCl; (**d**) with 0.1 M NaCl; (**e**) 0.5 M Na_3_C_6_H_5_O_7_; and (**f**) 0.1 M Na_3_C_6_H_5_O_7_.

Figure 7 shows the DSC thermogram of PUL powder and five PUL fibrous mats electrospun from 8% PUL solutions with various amounts of NaCl. It is clear that PUL sample *a* (powder before spinning) shows a higher melting temperature *T*_m_ at 144.5 °C than the electrospun sample *b* at 132.7 °C. The *T*_m_ of the PUL samples were further decreased by the inclusion of NaCl or Na_3_C_6_H_5_O_7_. As salt concentration increased, lower *T*_m_ were observed. The decrease in melting point is thought to reflect the change in PUL crystallinity. A rapid solidification process as the jet streams fly to and lie on the receptor surfaces during electrospinning should play a role in PUL crystallization [34,35,36]. In addition, the influence of the inclusion of salt as an effective crystallite also cannot be ignored, the interruption of hydrogen bonding and changes in molecular entanglements also contribute to the reduction of crystallinity, particularly at high salt concentrations. The crystallization of PUL electrospun from salted solution will be discussed in detail in our next publication.

The effects of electrospinning and the inclusion of NaCl on molecular properties of the 8% PUL were also investigated. As shown in Table 2, the *M*_w_ and *R*_gz_ of the electrospun PUL fibers were lower than those before submission to electrospinning, and these two values were further reduced by the presence of NaCl in PUL solution. The lower *M*_w_ (~226 × 10^3^ g/mol) and *R*_gz_ (~17 nm) observed for the PUL with NaCl when compared to the PUL without salt (~330 × 10^3^ g/mol and ~35 nm, respectively) could be related with the disruption of the H-bonds due to salt inclusion as well as some polymer degradation during fiber formation. The slight degradation of PUL macromolecules that occurred during electrospinning could probably be attributed to the interactions between surface tension and the electrostatic force built up by the high voltage.

The value of the second virial coefficient (*a*) is the indicator of the density of monomeric residues within the PUL molecular pack. All the three PUL samples had an *a* value of around 0.7, exhibiting an expanded coil conformation after re-dissolving in aqueous solutions. The close *a* values in the range of 0.659–0.675 could indicate a similar molecular packing of PUL segments with and without salt addition; next to adequate solution properties such as electrical conductivity, surface tension and viscosity, the well packed PUL molecules could lead to the uniform fibers and fiber sizes seen in Figure 1C after solvent evaporation, despite the lower *M*_w_ and *R*_gz_ of 8% PUL with 1.0 M NaCl.

## 4. Conclusions

The inclusion of sodium salts in PUL solutions altered the existing hydrogen bonding between the polysaccharides and water, as well as the inter- and intra-macromolecular chains. At low PUL concentration, the addition of a small portion of sodium salts eliminated bead formation on electrospun PUL fibers. As the concentration of both PUL and salts increases, the fiber sizes became bigger and the appearances of the fibers changed due to the increase in both the surface tension and electrical conductivity of the solution. The inclusion of sodium salts in PUL solutions also resulted in a smaller *M*_w_ and lower *T*_m_ in comparison with those before electrospinning or electrospun without sodium salts.

## Figures and Tables

**Table 1:** Specific viscosities of PUL solutions with various NaCl or Na_3_C_6_H_5_O_7_ contents.

PUL wt %	NaCl, M	Na_3_C_6_H_5_O_7_, M	Specific viscosity, Pa·s
15	0	0	204 ± 16
15	0.20	0	256 ± 21
15	2.0	0	333 ± 14
15	5.0	0	157 ± 17
15	0	0.05	237 ± 25
15	0	0.50	274 ± 32
8	0	0	19 ± 7
8	0.20	0	27 ± 2
8	1.0	0	36 ± 3
8	2.0	0	43 ± 11
8	0	0.05	22 ± 9
8	0	0.50	29 ± 4

**Table 2:** Molecular characteristics of 8% pullulan.

Submission to electrospinning	*M*_w_/*M*_n_	*M*_w_ × 10^−3^ (g/mol)	*R*_gz_ (nm)	*a* (mol.L/g)
Before	2.60 ± 0.03	360 ± 6	42.2 ± 1	0.672 ± 0.003
After	2.28 ± 0.02	330 ± 1	34.7 ± 1	0.663 ± 0.004
With 1.0 M NaCl	1.86 ± 0.02	226 ± 2	17.2 ± 1	0.668 ± 0.002
